# Intravenous lipid emulsion therapy for acute clomipramine intoxication in rats

**DOI:** 10.1002/ams2.344

**Published:** 2018-05-17

**Authors:** Takumi Tsuji, Yuji Hattori, Koji Komori, Yuya Yoshida, Rie Banno, Takeyuki Kohno

**Affiliations:** ^1^ Department of Pathological Biochemistry Faculty of Pharmaceutical Sciences Setsunan University Hirakata Osaka Japan; ^2^ National Hospital Organization Minami Wakayama Medical Center Wakayama Japan; ^3^ Department of Pharmacy Practice and Sciences Faculty of Pharmaceutical Sciences Setsunan University Hirakata Osaka Japan

**Keywords:** Clomipramine, intralipos, intravenous lipid emulsion therapy, lifesaving treatment, lipophilic drugs

## Abstract

**Aim:**

In this study, to assess the utility of lipid emulsion (ILE) therapy as a treatment option for overdoses of lipophilic drugs, we examined the detoxification effect of ILE therapy in rats that were administered overdoses of the tricyclic antidepressant clomipramine hydrochloride (CMI).

**Methods:**

Female Wistar rats were orally administered 50 mg/kg CMI five times in 2‐h intervals to examine whether intralipos accelerated the elimination of CMI in the peripheral blood. Rats were divided into the intralipos (i.v. 2 g/kg intralipos) and placebo (i.v. saline) groups. The concentrations of CMI and desmethylclomipramine (DMCMI), a metabolite of CMI, in blood were measured over time by high‐performance liquid chromatography. We then gave the animals 100 mg/kg CMI orally to examine whether intralipos could inhibit the distribution of CMI. The CMI and DMCMI concentrations in peripheral blood, liver, and brain were measured 60 min after intralipos administration.

**Results:**

The blood concentration of CMI was significantly higher in the intralipos group than in the placebo group at 60 and 120 min. After a single administration of 100 mg/kg CMI, the ratio of the concentration of CMI in liver/serum was significantly lower in the intralipos group than in the placebo group. We also found a significantly faster elimination rate for CMI in peripheral blood in the intralipos group than in the placebo group.

**Conclusion:**

The distribution of CMI from blood to tissue was suppressed by intralipos. Therefore, ILE therapy is a promising candidate for the treatment of overdoses of lipophilic drugs.

## Introduction

Intravenous lipid emulsion (ILE) therapy is a potentially lifesaving treatment of lipophilic drug intoxication. Weinberg *et al*.^1^ described how the administration of lipid emulsion decreased the cardiac toxicity of bupivacaine in rats. Weinberg *et al*.^2^ also reported that infusing a lipid emulsion during resuscitation from bupivacaine‐induced cardiac toxicity substantially improved hemodynamics and increased survival in dogs. The first report on the clinical application of ILE therapy was in 2006.^3^ Recently, ILE therapy has become a first‐line therapy for local anesthesia intoxication. Matsumoto *et al*.^4^ described how ILE therapy returned spontaneous circulation in a patient with lipophilic drug‐related cardiopulmonary arrest and reduced the blood concentration of the lipophilic drug. Chai *et al*.^5^ suggested that ILE therapy did not affect cocaine‐induced cardiac arrest in a rat model. In summary, evidence on the usefulness and safety of ILE for the therapy of lipophilic drug overdoses remains limited.

The leading hypothesis for the functional mechanism of ILE therapy is the “lipid sink hypothesis”, that is, intravascular lipid emulsion pulls the lipophilic drug back from the tissue into the blood vessel. Furthermore, uncertain evidence in support of this theory suggests that lipids can reverse both neurologic and cardiac toxicity, although the brain does not metabolize fatty acids as an energy source to an appreciable degree.^6^ Therefore, there are still some uncertainties surrounding the actions of lipophilic drugs once they are administered as a lipid emulsion. Winek *et al*.^7^ described how intoxication of clomipramine hydrochloride (CMI), a tricyclic antidepressant, is not fatal at any blood level. However, Okayasu *et al*.^8^ described how the use of tricyclic antidepressants prolonged the QTc interval. Therefore, CMI was our first choice for a lipophilic drug. An overdose of CMI can have fatal side‐effects (arrhythmia, QTc extension, QRS duration, or torsade de pointes). First‐line therapy is crucial as these side‐effects develop during the acute and not the chronic phase.

In this study, our goal is to assess the effectiveness of the ILE, intralipos, for treating CMI overdoses in a rat model.

## Methods

### Animals and ethics

Female Wistar rats weighing 170–200 g were purchased from Japan SLC (Shizuoka, Japan). The rats were bred and maintained under conventional conditions (23 ± 1°C and 47–67% humidity, under a 12:12‐h light : dark cycle) and accessed food (RCF‐1; Oriental Bio Co., Kyoto, Japan) and water ad libitum.

### Drugs

The CMI was purchased from Wako Pure Chemical Industries (Osaka, Japan). Intralipos injection 20% was purchased from Otsuka Pharmaceutical Factory. (Tokushima, Japan). Desmethylclomipramine (DMCMI), a metabolite of CMI, was purchased from Toronto Research Chemicals (Toronto, Canada).

### Study protocol

This study was composed of three separate experiments. For the first experiment, we induced an intoxication state in female Wistar rats based on the report of Weigmann *et al*.,^9^ as follows. Rats were given 50 mg/kg CMI orally five times in 2‐h intervals. The animals were divided into the intralipos (*n* = 6) and placebo (*n* = 4) groups. The intralipos group received i.v. intralipos (2 g/kg as soybean oil), and the placebo group received i.v. saline. Peripheral blood samples were collected after 0, 60, 120, 180, and 240 min.

The second experiment examined whether ILE therapy suppressed the tissue distribution of CMI. Female Wistar rats were treated once with oral 100 mg/kg CMI. The rats were again divided into the intralipos (*n* = 5; i.v. 2 g/kg intralipos as soybean oil) and placebo (*n* = 5; saline) groups. Peripheral blood and tissue samples were collected after 60 min.

In the third experiment, we assessed whether ILE therapy enhanced the elimination of CMI and DMCMI from peripheral blood. Female Wistar rats were administered i.v. 20 mg/kg CMI and divided into the intralipos (*n* = 3; i.v. 2 g/kg intralipos as soybean oil) and placebo (*n* = 3; saline) groups. Peripheral blood samples were collected after 0, 30, 60, 90, and 120 min.

### Serum and tissue extract preparation

Clomipramine hydrochloride and DMCMI were extracted from serum and tissue (of the brain and liver) based on previous reports.^9^, ^10^ Serum, brain, and liver samples were stored at −20°C until analysis.

For serum samples, an aliquot (15 μL) of the serum sample was mixed with 52.5 μL ethanol; subsequently, an internal standard (IS; 7.5 μL ethanol solution of 20 μg/mL cisapride) was added. An extract was obtained by centrifugation for 10 min at 8,400 ×g at 4°C, and 10 μL of the supernatant was used for analysis by high‐performance liquid chromatography (HPLC).

For tissue samples, the tissues were first thawed and 100 mg cortex or 20 mg liver was mixed with 350 μL ethanol and 15 μL IS. Subsequently, the samples were homogenized in the tube at 4°C with a homogenization pestle. After homogenization, an extract was obtained based on the methods of Aitchison *et al*.^10^ The extract was obtained by centrifugation for 10 min at 330 ×g at 4°C; subsequently, 200 μL supernatant was centrifuged again for 10 min at 14,200 ×g at 4°C, and 10 μL of this supernatant was used for analysis by HPLC.^10^

### High‐performance liquid chromatography

We used an HPLC system (JASCO Corporation, Tokyo, Japan) consisting of a model PU‐980 pump, AS‐950 injector, LCSS‐905 system controller, and UV‐970 detector set at 270 nm. Clomipramine hydrochloride and DMCMI were separated with a COSMOSIL 5C18‐AR‐II Paced column (4.6 × 250 mm; Nacalai Tesque, Kyoto, Japan) using a mobile phase consisting of 50% acetonitrile, 50% water, 0.1% trifluoroacetic acid, and 0.01% triethylamine. The mobile phase flow rate was set at 1.0 mL/min.

### Calibration

To create CMI and DMCMI calibration curves, standard samples (CMI and DMCMI at concentrations of 0, 5, 10, 25, 50, and 100 μg/mL in blank serum) were prepared. All standard samples underwent the extraction procedure described above. Calibration curves were obtained by using the peak ratios of the concentrations of CMI/IS or DMCMI/IS versus CMI or DMCMI, respectively. The CMI or DMCMI concentrations in each sample were calculated using these calibration curves.

### Recovery

The recovery rates of CMI and DMCMI from spiked samples were calculated from the peak height obtained by the extraction of serum at concentrations of 10, 25, and 50 μg/mL.

### Intra‐ and inter‐assay variation

Intra‐assay variations for CMI and DMCMI were determined by repeat analysis (five times) of quality control serum samples at concentrations of 10, 25, and 50 μg/mL on the same day. Inter‐assay variations were determined by repeat analysis of quality control serum samples at concentrations of 10, 25, and 50 μg/mL on five different days.

### Statistical analyses

Data were expressed as the mean ± standard deviation. The significance of the differences in CMI and DMCMI concentrations were evaluated using the Mann–Whitney *U*‐test. *P* < 0.05 was considered statistically significant. The statistical analysis software Statcel3 (OMS Publishing, Saitama, Japan) was used.

## Results

### High‐performance liquid chromatography analysis and validation

**Figure 1:**
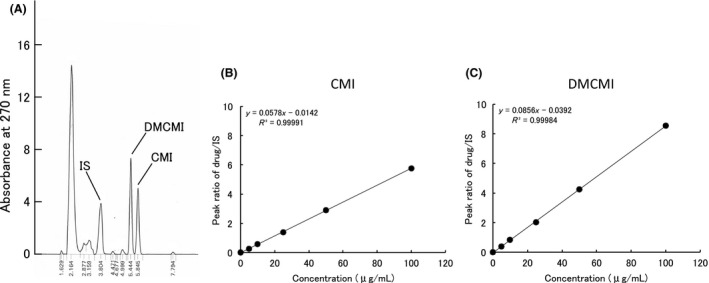
Representative chromatogram for clomipramine hydrochloride (CMI) and desmethylclomipramine (DMCMI) (A) and their respective standard curves (B, C). IS, internal standard.

In blank serum, no interfering peaks were detected at the retention times of CMI and DMCMI. The retention times of CMI, DMCMI, and the IS were 5.8, 5.4, and 3.8 min, respectively. A representative chromatogram is shown in Figure 1A. Standard curves were constructed by plotting the peak height ratios of CMI and DMCMI to those of the IS. The standard curve for CMI showed good linearity within the examined concentration range (5–100 μg/mL), had an *R* ^2^ value of 0.9999, and a regression equation of *y* = 0.0578*x* − 0.0142 (Fig. 1B). The standard curve for DMCMI also showed good linearity within the examined concentration range (5–100 μg/mL). It had an *R* ^2^ value of 0.9998 and a regression equation of *y* = 0.0856*x* − 0.0392 (Fig. 1C). Recovery of CMI and DMCMI was 98–116% (data not shown). The intra‐ and inter‐assay variations of CMI and DMCMI are shown in Table 1. The coefficients of variation for CMI and DMCMI were 4.7–5.6% and 4.7–14.4% for the intra‐ and inter‐assay variation, respectively.

**Table 1:** Intra‐assay (A) and inter‐assay (B) variability for clomipramine hydrochloride (CMI) and desmethylclomipramine (DMCMI)

CMI				DMCMI			
Conc. (μg/mL)	Experimental conc. (μg/mL)	SD	CV (%)	Conc. (μg/mL)	Experimental conc. (μg/mL)	SD	CV (%)
(A) Intra‐assay variation							
10	11.1	0.6	5.0	10	11.6	0.5	4.7
25	25.7	1.3	5.1	25	26.3	1.5	5.6
50	49.3	2.6	5.3	50	50.4	2.7	5.3
(B) Inter‐assay variation							
10	10.5	1.5	14.4	10	11.2	1.2	10.7
25	24.4	2.1	8.7	25	25.5	1.2	4.7
50	49.3	6.4	12.9	50	51.7	5.2	10.1

CMI, clomipramine hydrochloride; Conc., concentration; CV, coefficient of variation; DMCMI, desmethylclomipramine; SD, standard deviation.

### Concentrations of CMI and DMCMI in serum and tissue

**Figure 2:**
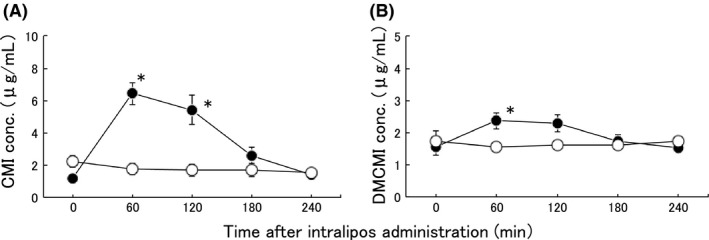
Concentrations of clomipramine hydrochloride (CMI) (A) and desmethylclomipramine (DMCMI) (B) in peripheral blood after repeat administration. Female Wistar rats were given 50 mg/kg CMI orally five times in 2‐h intervals. The animals were divided into the intralipos (●, *n* = 6) and placebo (○, *n* = 4) groups. The graphs depict means, with bars indicating standard errors. The significance of the differences between the intralipos and placebo groups was examined with the Mann–Whitney *U*‐test. *P < 0.05.

First, we measured CMI and DMCMI concentrations in serum and tissue. Whereas no peaks for CMI and DMCMI were detected in serum in the placebo group, well‐defined peaks were observed in the intralipos group (Fig. 2). Specifically, the CMI concentration in serum was significantly higher (*P* < 0.05) in the intralipos than in the placebo group at 60 min (6.4 ± 1.7 μg/mL versus 1.8 ± 0.8 μg/mL, respectively) and 120 min (5.4 ± 2.2 μg/mL versus 1.7 ± 0.8 μg/mL, respectively; Fig. 2A). For DMCMI, we also found a significantly higher (*P* < 0.05) concentration in serum in the intralipos than in the placebo group at 60 min (2.4 ± 0.7 μg/mL versus 1.6 ± 0.3 μg/mL, respectively; Fig. 2B).

### Effect of intralipos on the suppression of the distribution of CMI and DMCMI from blood to tissue

**Figure 3:**
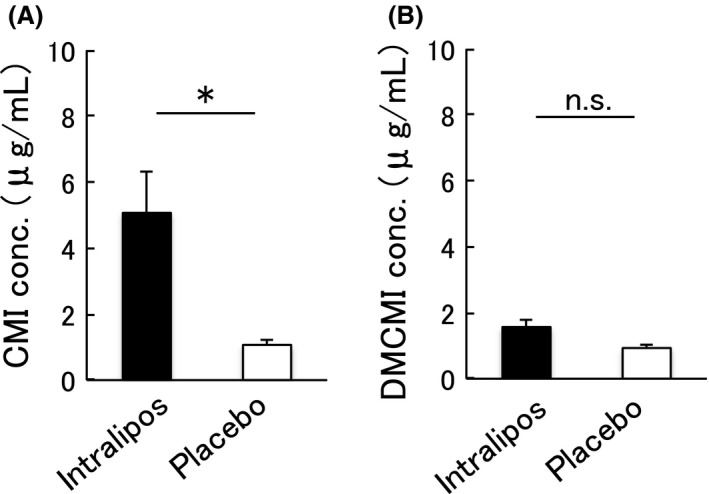
Concentrations (conc.) of clomipramine hydrochloride (CMI) (A) and desmethylclomipramine (DMCMI) (B) in peripheral blood after a single administration. Female Wistar rats were given 100 mg/kg CMI orally, and the animals were divided into the intralipos (*n* = 5) and placebo (*n* = 5) groups. CMI (A) and DMCMI (B) concentrations in peripheral blood 60 min after administration are shown. Means and standard errors (bars) are shown. The significance of the differences between the intralipos and placebo groups was examined with the Mann–Whitney *U*‐test. *P < 0.05.

**Figure 4:**
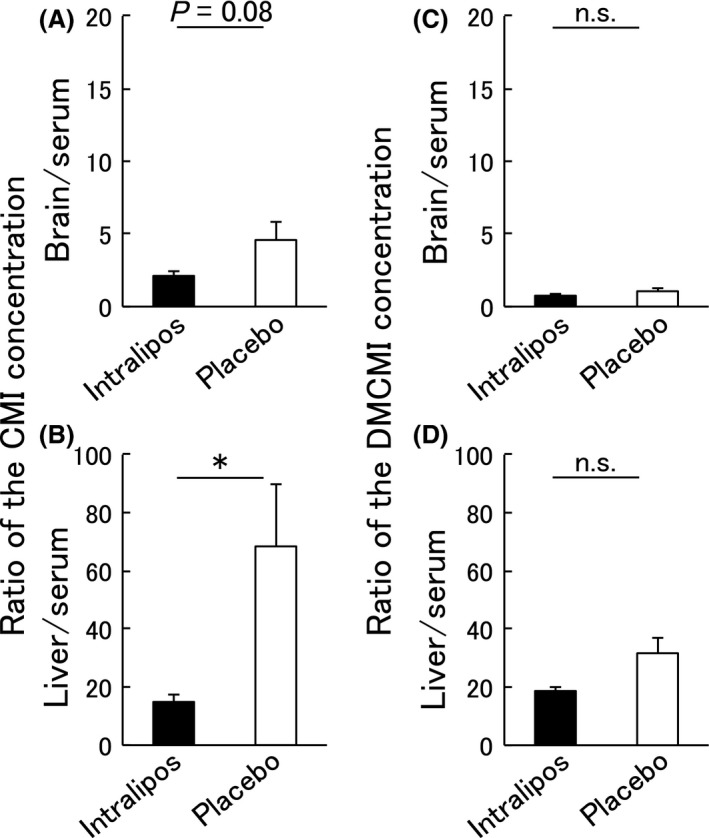
Ratios of tissue/serum concentrations of clomipramine hydrochloride (CMI) and desmethylclomipramine (DMCMI) in the brain and liver after a single administration. Female Wistar rats were given oral 100 mg/kg CMI or DMCMI orally, and the animals were divided into the intralipos (*n* = 5) and placebo (*n* = 5) groups. The ratios of the tissue/serum concentrations in the brain and liver 60 min after administration are shown for CMI (A, B) and DMCMI (C, D) in the brain and liver. Means and standard errors (bars) are shown. The significance of the differences between the intralipos and placebo groups was examined with the Mann–Whitney *U*‐test. *P < 0.05. n.s., not significant.

We then examined whether ILE could suppress the distribution of CMI and DMCMI from peripheral blood to the tissue (the second experiment). We found a significantly higher CMI concentration in serum in the intralipos group than in the placebo group (5.1 ± 2.5 μg/mL versus 1.1 ± 0.4 μg/mL, respectively; Fig. 3A). No significant differences between the two groups were observed for DMCMI (Fig. 3B). In the intralipos group, the ratio of the CMI concentration in tissue/serum was showed a tendency of being lower in brain samples (Fig. 4A) and significantly lower (P < 0.05) in liver samples (Fig. 4B) when compared to the placebo group. No significant differences in the ratios of the DMCMI concentration in tissue/serum were found between the two groups (Fig. 4C, D).

### Effect of intralipos on the elimination of CMI from peripheral blood

**Figure 5:**
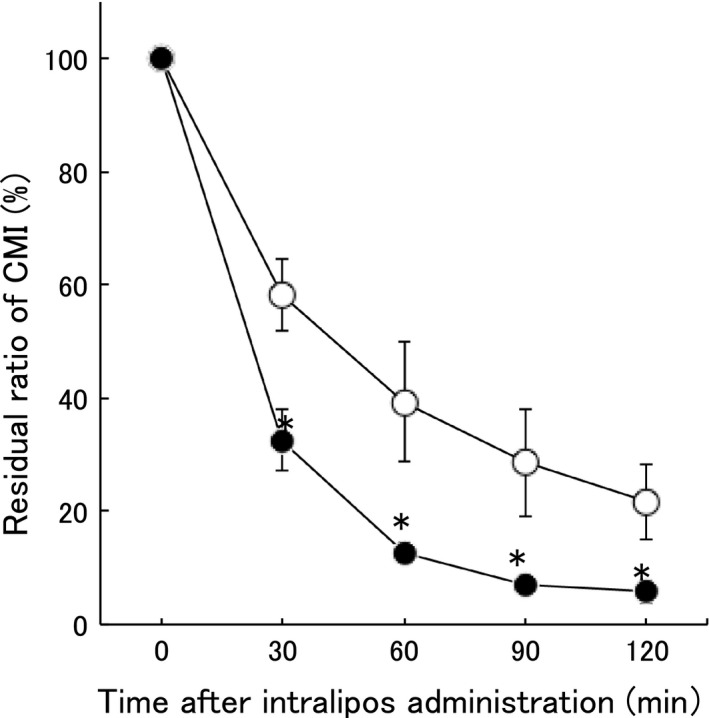
Residual ratio of clomipramine hydrochloride (CMI) in peripheral blood after i.v. administration. Female Wistar rats received 20 mg/kg CMI i.v. The rats were divided into the intralipos (●, *n* = 3) and placebo (○, *n* = 3) groups. The graph depicts the means, with bars indicating standard errors. The significance of the differences between the intralipos and placebo groups was examined with the Mann–Whitney *U*‐test. *P < 0.05.

Finally, we examined whether ILE enhanced the elimination of CMI from peripheral blood. We found a significantly faster (*P* < 0.05) elimination rate in the intralipos group when compared to the placebo group (Fig. 5).

## Discussion

In the present study, we make it clear that ILE therapy (i.v. administration of intralipos) suppressed the distribution of CMI from blood to tissue and enhanced its elimination from peripheral blood.

For the lipid emulsion preparation, a 20% injection product of 250 mL is commonly used as a caloric and essential fatty acid supplement. The primary component of intralipos is soybean oil, but the drug also includes linolenic acid, oleic acid, and palmitic acid. Intralipos should not be given to patients with other than thrombosis, severe hepatopathy, severe coagulopathy, dyslipidemia, and ketosis from diabetes. However, the safety and effectiveness of ILE therapy for acute poisoning with lipophilic drugs are poorly understood.

In the first experiment (repeat administration), the CMI and DMCMI concentrations in serum were significantly higher in the intralipos group than in the placebo group. In the second experiment (single administration), we found a significantly higher CMI concentration in serum in the intralipos group than in the placebo group. In contrast, the ratio of the CMI concentration in tissue/serum concentration was lower in the intralipos than in the placebo group. These findings suggest that ILE therapy inhibited the distribution of CMI to the organs. The third experiment revealed that the elimination of CMI in peripheral blood was significantly faster in the intralipos than in the placebo group. This finding suggests that ILE therapy promoted the elimination of CMI. Of note, the DMCMI concentration was lower than the CMI concentration, therefore the behavior of DMCMI was different than that of CMI. Additionally, it has been suggested that the difference in CMI concentrations between the brain and liver tissue had an overall positive effect on the distribution of CMI to peripheral tissues. Litonius *et al*.^11^ also reported a similar finding in their study on acute poisoning with bupivacaine.

Although we need to expand the sample size, our data suggest that intralipos can be used to treat acute CMI overdoses. The proof of the usefulness of ILE therapy will likely result in the increased use of this therapeutic strategy (e.g., for overdoses of other lipophilic drugs). In a future study, we aim to show that ILE therapy is effective as a lifesaving treatment of overdoses of other lipophilic drugs.

## Disclosure

Approval of the research protocol: This study was carried out according to a protocol approved by the Institutional Animal Care Committee of Setsunan University.

Informed consent: Not applicable.

Registry and the registration no. of the study/trial: This study was registered in the Setsunan University, nos. K15‐16 and K16‐17.

Animal studies: Throughout the experimental procedures, every effort was made to minimize animal suffering and the number of animals used. This experimental procedure was approved by the Institutional Animal Care Committee of Setsunan University.

Conflict of interest: None declared.
